# Functional characterization of three MicroRNAs of the Asian Tiger Mosquito, *Aedes albopictus*

**DOI:** 10.1186/1756-3305-6-230

**Published:** 2013-08-08

**Authors:** Santhosh Puthiyakunnon, Yunying Yao, Yiji Li, Jinbao Gu, Hongjuan Peng, Xiaoguang Chen

**Affiliations:** 1 Key Laboratory of Prevention and Control for Emerging Infectious Diseases of Guangdong Higher Institutes, Department of Pathogen Biology, School of Public Health and Tropical Medicine, Southern Medical University, Guangzhou North Avenue No.1838, Guangzhou 510515, China

**Keywords:** *Aedes albopictus*, microRNA, Mosquito, Longevity, Fecundity, Eclosion, Hatching rate, Microinjection

## Abstract

**Background:**

Temporal and stage specific expression of microRNAs (miRNAs) in embryos, larvae, pupae and adults of *Aedes albopictus* showed differential expression levels across the four developmental stages, indicating their potential regulatory roles in mosquito development. The functional characterization of these miRNAs was not known. Accordingly our study evaluated the functional characterization of three miRNAs, which are temporally up-regulated in the various developmental stages of *Ae. albopictus* mosquitoes.

**Methods:**

miRNA mimics, inhibitors and negative controls were designed and their knock-in and knock-down efficiency were analyzed by qRT-PCR after transfecting the mosquito cell lines C6/36, and also by injecting in their specific developmental stages. The functional role of each individual miRNA was analyzed with various parameters of development such as, hatching rate and hatching time in embryos, eclosion rate in larvae, longevity and fecundity in the adult mosquitoes.

**Results:**

The knock-in with the specifically designed miRNA mimics showed increased levels of expression of miRNA compared with their normal controls. We confirmed these findings using qRT-PCR, both by *in vitro* expression in C6/36 mosquito cell lines after transfection as well as in *in vivo* expression in developmental stages of mosquitoes by microinjection. The knock-down of expression with the corresponding inhibitors showed a considerable decrease in the expression levels of these miRNAs and obvious functional effects in *Ae. albopictus* development, detected by a decrease in the hatching rate of embryos and eclosion rate in larvae and a marked reduction in longevity and fecundity in adults.

**Conclusion:**

This study carried out by knock-in and knock-down of specifically and temporally expressed miRNAs in *Ae. albopictus* by microinjection is a novel study to delineate the importance of the miRNA expression in regulating mosquito development. The knock-down and loss of function of endogenously expressed miRNAs by the miRNA inhibitors in specific developmental stages had considerable effects on development, but enhancement of their gain of function was not observed on knock-in of these specific miRNAs. Hence, our study indicates that an optimal level of endogenous expression of miRNA is indispensable for the normal development and maintenance of the vectorial population density and pathogen transmissibility of this mosquito vector.

## Background

Mosquito-borne diseases like, Dengue fever, malaria, yellow fever and other encephalitis viral infections are widely prevalent and the high mortality caused in humans and animals in many tropical countries drives the need for control of these diseases. Despite many traditional control measures, their failure has sparked interest in several new approaches [1,2]. These include the development of genetically modified mosquitoes (GMMs) designed either to reduce population sizes or to replace existing populations with vectors unable to transmit the disease [3,4]. One new approach which is in the initial stages of development has emerged with the understanding of newly discovered RNAs like microRNA (miRNA) and other short RNAs which have immense capacity to function as regulatory molecules in many essential biological functions in humans [5,6], plants [7] and all living species including insects [8,9] which are important vectors for spreading many diseases.

MiRNAs are an abundant group of newly identified endogenous non-protein coding small RNAs of 20–25 nucleotides [10,11]. Currently, miRNAs have been considered one of the most important regulatory molecules, which regulate gene expression at the posttranscriptional levels by targeting mRNAs for direct cleavage of mRNAs or repression of mRNA translation [6,12,13]. Many studies have explored the regulatory role of miRNA in different stages of *Drosophila melanogaster* and have successfully established their biological importance in other insects including various mosquito species acting as vectors for transmitting diseases [8,14]. Differential gene expression and proteomic studies have contributed significantly to our understanding of developmental, ecological and behavioral changes in insects [15,16].

The Asian tiger mosquito, *Aedes albopictus* (Skuse, 1894), is an invasive species that can be currently found in temperate and tropical Asia (its area of origin), Europe, North and South America, Africa and a number of locations in the Pacific and Indian Oceans [17]. In China, the first outbreak of dengue was confirmed in Guangdong province in 1978. Since then, dengue epidemics were reported sequentially in Hainan, Guangxi, Fujian, Zhejiang and Yunnan provinces [18]. *Ae. albopictus,* a peridomestic mosquito was recently identified as the only known vector of dengue in the Pearl River Delta area in Guangdong Province [19,20], which has displaced *Aedes aegypti*, the fully domestic dengue vector [21-23]. This further confirms the importance of a surveillance system and a prompt reaction to control the diseases spreading, such as controlling vector density of this recently emerged mosquito vector, *Ae. albopictus*.

Quantitative PCR and microarray are promising tools to understand the expression patterns and functions of miRNAs [6,24,25]. Recently our lab identified a total of 140 *Ae. albopticus* mature miRNAs in which 91 were conserved across species and 49 turned out to be *Aedes* specific [26]. The miRNAs were identified for the first time, in *Ae. albopictus*, which is the major vector of dengue fever in China and its genome sequence is not known. Temporal and stage specific expression of miRNAs in eggs, larvae, pupae and adults (sugar fed males and blood fed females) were done by a high throughput Solexa sequencing and was confirmed by Northern blot analysis. Our results showed an apparently different expression level of these miRNAs across the four developmental stages of *Ae. albopictus* indicating their potential roles in the regulation of mosquito development (Table 1).

**Table 1:** **Differential expression of three miRNAs (copy number) in *Ae. albopictus *developmental stages; embryo, larvae, pupae and adults (male and female)**

**miRNA name**	**Embryo**	**Larvae**	**Pupae**	**Male**	**Female**
aal-miR-1891	6	2	10	7658	12518
aal-miR-286b	18703	9	1	0	3
aal-miR-2942	23	3044	450	43	38

Here, we describe a study of the three specific miRNAs, which are temporally and specifically up-regulated in each developmental stages of *Ae. albopictus* to delineate the importance of the miRNA’s in the development and survival of this important mosquito vector species. We have specifically knocked-in and knocked-down the miRNA expression in the developmental stages; embryo, larvae and adults with the specific miRNA mimic and inhibitors and analyzed the various growth and biological development of this vector. Through this novel study, we unravel here the functional roles played by these miRNA’s in *Ae. albopictus,* an emerging mosquito vector species spreading the major viral diseases like dengue and chikungunya.

## Methods

### Sequence analysis of three important miRNAs up-regulated in each stage of *Ae. albopictus* mosquitoes

The functional miRNAs up-regulated or down-regulated in each developmental stage of mosquito specifically and temporally expressed in adult female, adult male, larvae, pupae and embryo stages were analyzed and three important miRNAs which were specifically up-regulated in adults, larvae and embryos were selected for further study. They are aal- miR-1891 in adults, aal-miR-286b in embryos and aal-miR-2942 in larvae. The mature miRNA and precursor miRNA sequence were obtained from the *Ae. aegypti* miR-base and were compared for sequence analogues and found that they are conserved among these two mosquito species (Additional file 1).

### Design of microRNA mimic, inhibitor and negative controls

The miRNA mimic will increase the function of endogenously expressed miRNA whereas miRNA inhibitors suppress their function by increasing the target gene expression. Similarly, the over expression of miRNAs by a miRNA mimic will improve the phenotypic change and knocking down with miRNA inhibitors will weaken the phenotypic expression by the corresponding miRNA. The miRNA mimic, inhibitor and negative controls were designed and procured from GenePharma (Shanghai GenePharma Co., Ltd) at a concentration of 20 μM each. The details of their sequences are included (Additional file 2).

### Cell cultures and transfection

C6/36 cells were maintained at 28°C in Dulbecco’s modified Eagle’s medium (DMEM, Invitrogen) supplemented with 10% fetal bovine serum (FBS), 100 units/mL penicillin, and 100 mg/mL streptomycin (Invitrogen). Cells were regularly passaged at sub-confluence and plated 24 hrs before transfection at 90% confluence. Lipofectamine 2000 (Invitrogen) mediated transfection of miRNAs (mimic, inhibitor, and negative controls) were performed in six-well flat bottom plates with lids (Costar Co.) [27]. A transfection mixture containing 100 pmol/μL miRNAs and 7 mL of lipofectamine in 1 mL of serum-reduced OPTI-MEM (Invitrogen) were added to each well. Cells were incubated in the transfection mixture for 4 hrs and further cultured in antibiotic-free DMEM. Normal cells without any treatment were served as mock controls.

### Reverse transcription and quantitative PCR (qRT-PCR)

The total RNA from the transfected cells and mosquito embryos, larvae and adults were extracted using the miRNA Isolation kit (Ambion, Life technologies). The concentration of total RNA was quantified by Nanodrop 2000 spectrophotometer (Thermo Scientific). The quality and purity of total RNA was detected by running electrophoresis in 1% agarose gel (Additional files 3 &4). Reverse transcription to make cDNA was done using the miScript Reverse Transcription kit (Qiagen). miRNA quantification was calculated by the Bulge-loop™ miRNA qRT-PCR method [28,29] and primer sets (one RT primer and a pair of qPCR primers for each set) specific for miR-1891, miR-286b and miR-2942 are designed by RiboBio (Guangzhou, China). The qRT-PCR was repeated three times to validate the experimental design and result.

### Mosquito maintenance

The *Ae. albopictus* strain used in this work was obtained from the Center for Disease Control and Prevention of Guangdong Province. Mosquitoes were reared at a temperature of 27°C and a relative humidity of 70 to 80% with 14/10 h light/dark cycles [27]. Larvae were reared in pans and fed on finely ground fish food mixed 1:1 with yeast powder. Mosquito adults were fed on a 10% sugar solution. Adult females between 3 and 4 days post-emergence were fed on anesthetized mice when a blood meal was required. Eggs for microinjection were collected by forced laying from females after 2 days post-blood feeding.

### Microinjection of embryo

Embryo microinjection was based upon techniques successfully used for mosquito transgenesis [30]. Approximately ten blood-fed *Ae. albopictus* females were held in Drosophila vials (Fisher Scientific) containing a wet filter paper funnel. Eggs were laid in a solution of 0.1 mM p-nitrophenyl p’-guanidinobenzoate (pNpGB) (Sigma) in isotonic buffer (150 mM NaCl, 5 mM KCl; 10 mM HEPES; 2.5 mM CaCl_2_; pH 7.2). Eggs were then left in pNpGB solution until microinjection, which was carried out ~60 min after oviposition. Following a brief desiccation, gray embryos were aligned on double sided tape (Scotch 665; St. Paul, MN) and covered with halocarbon oil 700 (Sigma-Aldrich Co.). Injection needles (Quartz with filament, O.D.: 1.0 mm, I.D.: 0.70 mm) were pulled with a P2000 micropipette puller (Sutter Instrument Co., Novato, CA). Embryos were injected in separate batches into the posterior pole with a mixture of miR-286b (mimic or inhibitor) in injection buffer (5 mM KCl, 0.1 mM sodium phosphate, pH 6.8) using an Eppendorf Micromanipulator & Transjector (model: Eppendorf AG 22331, Hamburg, Germany) which controls the injection volume, pressure and back flow following the protocols of the double sided sticky tape method [31-33]. After injection, the embryos were incubated at 80% relative humidity and 27°C for approximately 40 min. Embryos were then removed from the oil and transferred to wet filter paper and allowed to develop for 3 days. A set of control eggs were injected with a negative control and kept for hatching in parallel with a set of un-injected eggs laid in the same batch.

### Microinjection of adults and larvae

*Ae. albopictus* larvae and adult mosquitoes were microinjected using an air drawn syringe set up prepared in our laboratory [34,35]. Larvae of a second instar stage were injected in the thorax after slight anaesthetizing to immobilize by keeping in ice cold water for 1–2 minutes [36] and injected with miR-2942 mimic /inhibitor in separate batches. Adult mosquitoes were anaesthetized by CO_2_ and injected in the thorax with miR-1891 mimic and inhibitor in two separate batches. Both larvae and adults were injected each with 0.5 μl (500 nL) of miRNA (mimic /inhibitor) at a final concentration of 20 pmols /μL. Larvae were transferred to a new bowl of fresh water with the larval food and transferred to the insectary. Adult mosquitoes were transferred to small plastic cups immediately after injection and allowed to recover. A set of injection control were set up for both larvae and adults injected with the same volume of negative controls and also reared in parallel with normal larvae and adults as un-injected controls.

### Functional interventional studies

Stage specific expression of three miRNAs, miR-286b, miR-2942 and miR-1891, which are up-regulated in *Ae. albopictus* were further evaluated in this study to explore their importance in the development of this mosquito species. The functional roles of the miRNA expression in specific stages were studied by analyzing the key parameters of development in the mosquito life cycle such as, the hatching rate of embryos, the eclosion rate of larvae and longevity (life span) and fecundity (egg laying capacity) of the adult mosquito.

### Life span (longevity) and Egg laying capacity (fecundity) of adult *Ae. albopictus* with miR-1891 interventions

Adult *Ae. albopictus* mosquitoes were selected after 3 days post-emergence in 4 groups (mimic, inhibitor, negative control and un-injected normal) including approximately 40 individuals with an almost equal proportion of male and female. Three groups were subjected to microinjection with miRNA-1891- mimic, inhibitor and negative control at a final concentration of 20 pmols /μL and transferred to the insectary with the specified conditions. The survival time (longevity) of mosquitoes was recorded on a daily basis at least twice per day and a life table was created for each group separately and analyzed statistically. To evaluate the effect of miR-1891 on fecundity, adult *Ae. albopictus* mosquitoes were selected after 3 days of post emergence in four groups, each having 5 mosquitoes (3 males :2 females) and three groups were injected with miRNA-1891 -mimic, inhibitor and negative control at a final concentration of 20 pmols /μL and transferred to the insectary. Mosquitoes were fed with a 10% sugar solution twice a day and females were fed on anaesthetized mice after 4 days of microinjection and allowed to lay eggs in the egg laying module kept in each cage 2 days after blood feeding. Eggs were collected after two days of complete laying from each group and counted and statistically analyzed. A normal group of un-injected mosquitoes were also analyzed for egg laying in parallel.

### Hatching rate and Hatching time analysis of embryos with miR-286b intervention

Approximately 150 eggs were subjected to microinjection in each group with miR-286b-mimic, inhibitor and negative control according to the microinjection protocols and allowed to hatch separately in the insectary as per the hatching conditions mentioned above. The hatching rate was calculated for each group including the normal control group with un-injected eggs. Hatching rate and development of larvae to pupae and adults were determined for each group to record the percentage of successful complete development to the adult stage. The experiments were repeated three times on different days to validate the experimental result and statistically analyzed. The daily hatching of number of eggs were also recorded in each group and the effect of miRNA on normal hatching time was determined.

### Eclosion rate of larvae with miR-2942 intervention

Four groups of 10–20 larvae of the 2^nd^ instar stage were separated and three groups were subjected to microinjection with miR-2942 mimic, inhibitor and negative control according to the protocol mentioned above. The larvae were observed for growth and development to the adult stage. The eclosion rate in each group were determined and statistically analyzed including a parallel group of un-injected larvae. All the functional experiments were repeated three times at different time points to validate the results.

### Ethics statement

Mosquito blood-feeding on anaesthetized mice was authorized by Fiocruz Ethical committee (CEUA 007/009).

## Results and discussion

### Stage-specific mosquito microRNA expression

Based on a previous study of microarray analysis, the functional miRNAs up-regulated or down-regulated in each of the developmental stages of mosquito specifically expressed at female adult, male adult, larvae, pupae and embryo stages were analyzed and three important miRNAs, which were specifically up-regulated in adults, larvae and embryo were selected for our study. The three miRNAs are aal- miR-1891 in adults, aal-miR-286b in embryos and aal-miR-2942 in larvae (Table 1). Increased expression of miR-286b only in the embryo clearly suggests its important role in embryogenesis and metamorphosis. miR-2942 was normally expressed at the egg, pupae and adult stages; however, it had the highest level of expression in larvae. Furthermore, miR-1891 was specifically expressed in high copy number in adults, and comparatively higher levels in females suggest their possible regulatory role in blood feeding and egg development. This dramatic alteration of expression during these three stages signifies that these three miRNA may be involved in the regulation of mosquito development.

### qRT-PCR of miRNA expression after miRNA intervention in C6/36 cells (*in vitro*) and in *Ae.albopictus* stages (*in vivo*)

**Figure 1:**
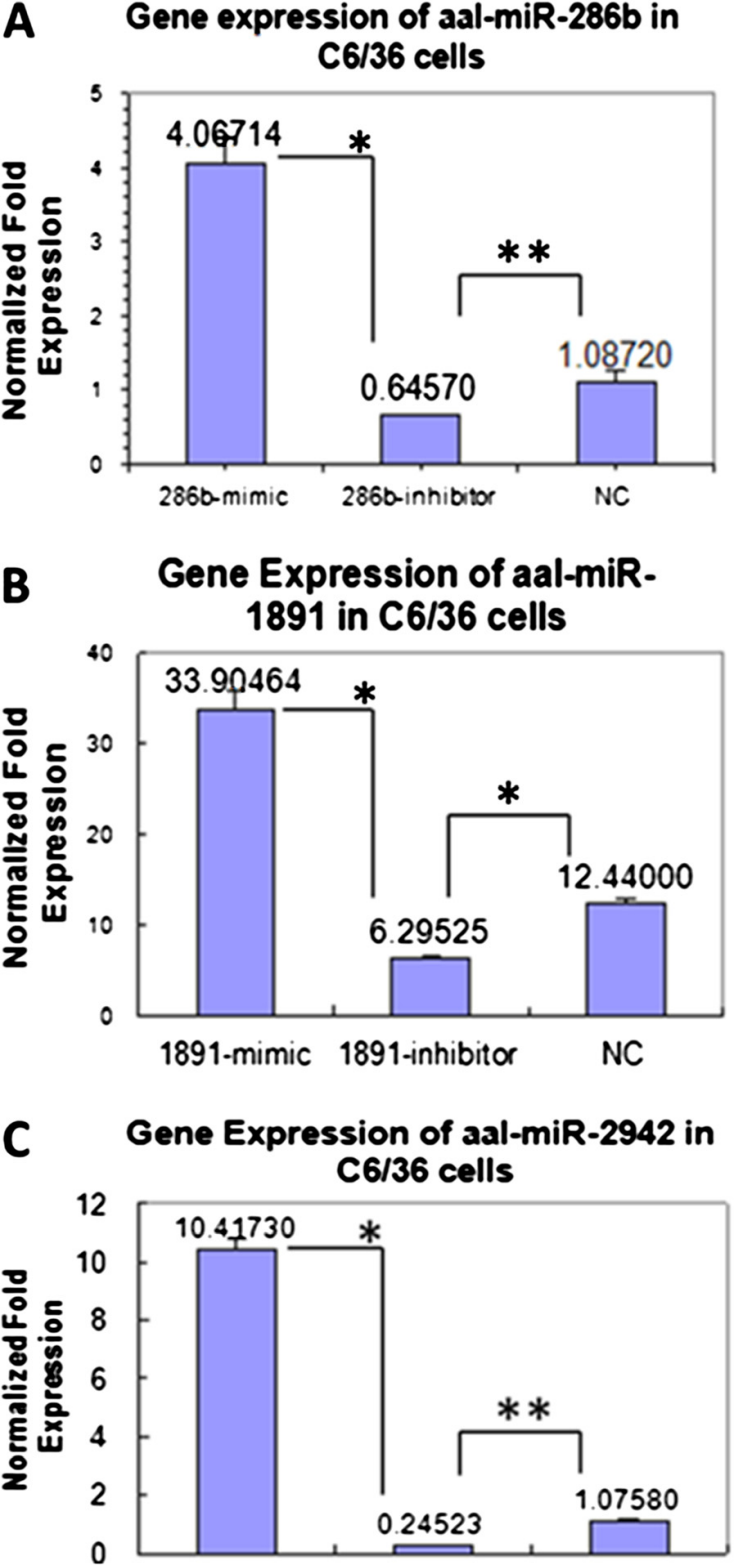
**Relative expression analysis after miRNA interference with three miRNAs in C6/36 cells by Bulge-loop miRNA qPCR. (A)** Relative expression of miR-286b analyzed by ANOVA (F = 72.047, p < 0.001). **(B)** Relative gene expression of miR-1891 analyzed by ANOVA (F = 146.96, p < 0.001). **(C)** Relative expression of miR-2942 analyzed by ANOVA (F = 496.035, p < 0.001). * showed a significant decrease (p < 0.001) in expression level in inhibitor group compared with the negative control group (analyzed by statistical method of ANOVA by LSD). ** No significant difference (p > 0.05) in expression level between inhibitor and negative control group (analyzed by statistical method of ANOVA, LSD). In all qPCR quantification, the relative gene expressions from normal controls are calibrated to 1 (not shown in graph). Error bars show the SEM of three biological replicates from three independent cell transfection experiments.

**Figure 2:**
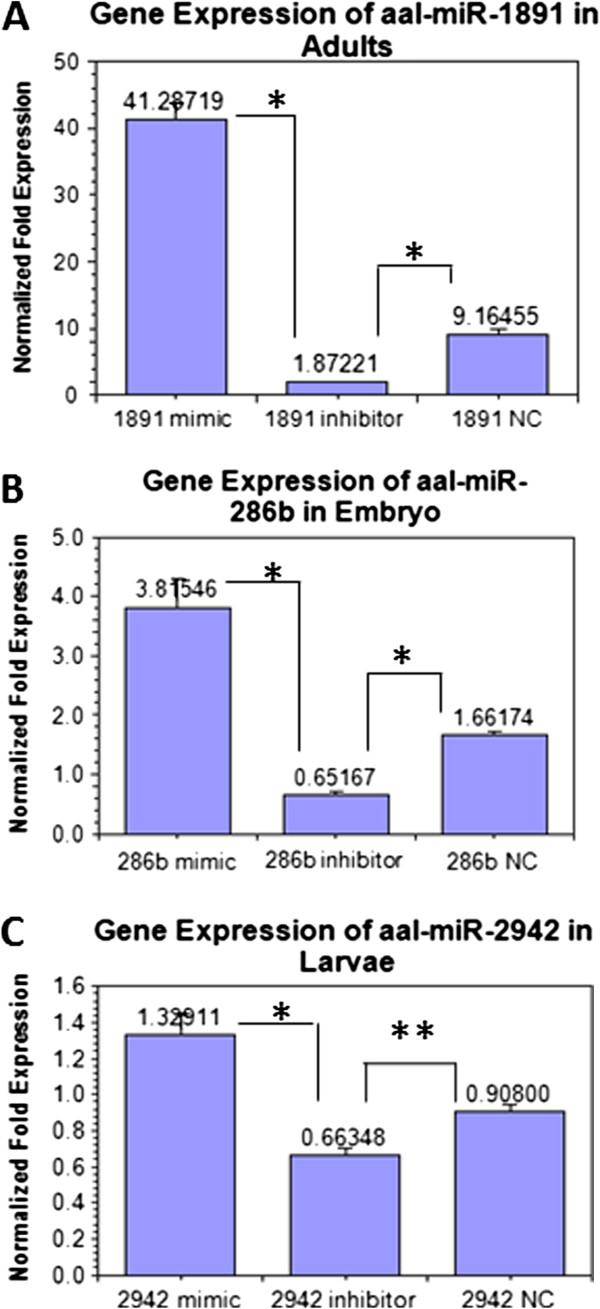
**Relative expression analysis after miRNA interference with three miRNAs in developmental stages of *Ae. albopictus *by Bulge-loop miRNA qPCR. (A)** Relative gene expression of miR-1891 in adults analyzed by ANOVA (F = 180.22, p < 0.001) **(B)** Relative expression of miR-286b in embryo analyzed by ANOVA (F = 32.908, p < 0.001) **(C)** Relative expression of miR-2942 in larvae analyzed by ANOVA (F = 19.648, p = 0.002). * A significant decrease (p < 0.001) of expression in inhibitor group compared with the negative control group (analyzed by statistical method of ANOVA by LSD). ** No significant difference (p > 0.05) in expression level between inhibitor and negative control group (analyzed by statistical method of ANOVA, LSD). In all miRNA qPCR quantifications, the relative gene expressions from normal controls are calibrated to 1 (not shown in graph). Error bars show the SEM of three biological replicates, each containing one adult male/female.

The relative expression of three miRNAs after their knock-in and knock-down with their mimics and inhibitors in the insect cell line C6/36, were quantified by Bulge loop miRNA qRT-PCR (Figure 1A-C). The relative expression of three miRNAs after miRNA intervention with mimics and inhibitors were also performed in specific developmental stages of *Ae. albopictus* and quantified by Bulge loop miRNA qRT-PCR (Figure 2A-C). The U6 primers were used as the controls in both *in vitro* and *in vivo* expression. A significant level of knock-in and knock-down efficiency was shown by three miRNA mimics and inhibitors against their miRNA expression in specific developmental stages; embryos, larvae and adults of *Ae. albopictus*.

### Functional interventional studies of mosquito miRNAs

The specific functional analysis of the three miRNAs in the developmental stage where they were over-expressed was performed. As the genome sequencing of *Ae. albopictus* is yet to be done, the specific role of each miRNA for its target proteins and its specific translational inhibition to reveal the molecular role played by this miRNA could not be achieved. Hence the overall developmental functions such as the hatching rate of embryos, the eclosion rate of larvae and egg laying capacity and life span of adults were studied as indicators of the functional role of each of these stage specific miRNAs in mosquito development.

### miR-286b intervention in *Ae. albopictus* embryos

**Figure 3:**
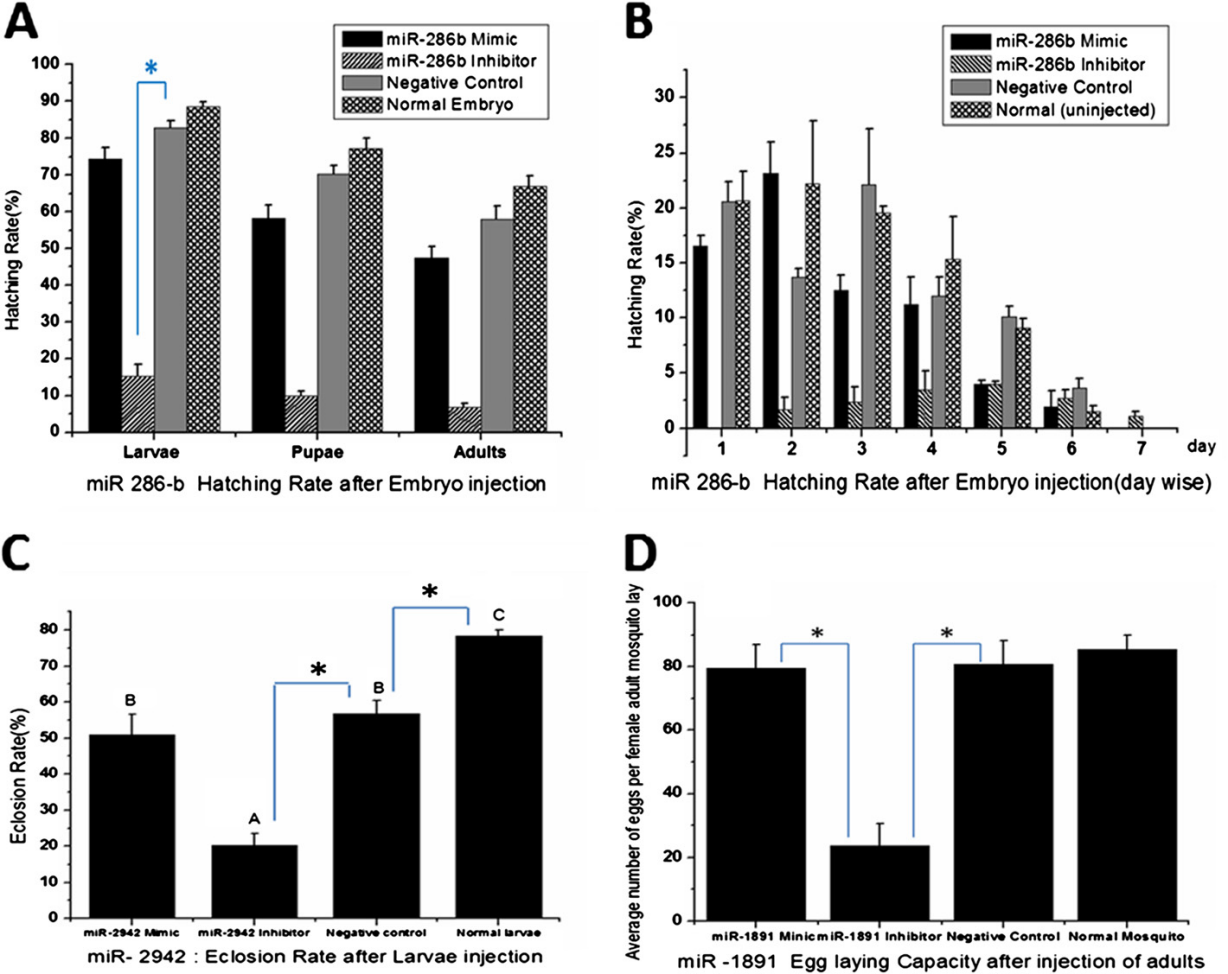
**Functional study of miRNA intervention after miR-mimic and inhibitor injection in *Aedes albopictus *developmental stages. [A]** Hatching rate of embryo after miR-286b injection with mimic, inhibitor, negative control and normal un-injected embryos. * A significant difference (p < 0.001) of inhibitor group with the negative control group in hatching rate of eggs into larvae (statistically analyzed by ANOVA, LSD). The error bar shows the SD of values from three independent experiments, and is statistically evaluated by ANOVA (F = 191.557 & p < 0.001). **[B]** Daily hatching after embryo injection with miR-286b mimic, inhibitor, negative control and normal un-injected embryo. The 1^st^ hatching day is 2 days after the microinjection. The error bar shows the SD of values from three independent experiments. **[C]** Eclosion rate in larvae after miR-2942 injection with mimic, inhibitor, negative control and normal un-injected embryo analyzed statistically by ANOVA (F = 38.193 & p < 0.001). * A statistically significant difference (p < 0.001) of inhibitor group with the negative control group and other two groups (statistically analyzed by ANOVA, LSD). A, B, C indicates statistically significant difference (p < 0.001) between the groups (statistically analyzed by ANOVA, LSD). The error bar shows the SD of values from three independent experiments. **[D]** Fecundity rate after miR-1891 injection. Result analyzed by ANOVA (F = 58.886 and p < 0.001). *A significant decrease in mean eggs per female in inhibitor group (p < 0.001) compared with negative control group (statistically analyzed by ANOVA, LSD) and all other groups observed. The error bar shows the SD of values from three independent experiments.

Hatching rate of embryos after miR-286b intervention showed a profound decrease in the number of embryos that hatched after knock-down of miR-286b expression (p < 0.001). This knock-down effect was carried throughout till the development to adults as indicated by the reduced number of larvae developed to pupae and adults in this group. The percentage of mean hatching rate decreased from 74.43% in mimic group to 15.28% in inhibitor group (analyzed by ANOVA, F =191.557 & p < 0.001, Figure 3A).

The miR-286b mimic group did not show any elevated hatching rate, which indicates a saturation level maintained by this induced miRNA expression in embryos. These findings clearly indicate that the endogenous expression of miR-286b is indispensable for the normal hatching of embryos and further development in the *Ae. albopictus* life cycle. The negative control (injection control) and normal embryo group (un-injected control) showed no significant difference (p = 0.128), which indicates the technical success of microinjection of embryo.

A further evaluation of temporal expression related to miRNA and its exact role played in embryo hatching was studied by careful analysis of daily hatching rates of injected embryos. The embryo hatching time has shown a direct relationship with the miR-286b expression. The inhibitor group showed a remarkable delay in most of its embryo hatching time, compared to other groups (the normal time taken for hatching after the eggs are put in water is 2–3 days). A delay in hatching may be one of the reasons for the reduced hatching success. A considerable delay in hatching of most of the eggs from inhibitor group in first three days was noticed, whereas most of the eggs (nearly 80%) from other three groups hatched during this period (Figure 3B).

### miR-2942 intervention in *Ae. albopictus* larvae

The mean eclosion rate of larvae in the inhibitor group (20.26%) where miR-2942 expression was knocked-down showed a significant decrease (p < 0.001) compared to the mimic group (50.9%) and also with the other two control groups (analyzed by ANOVA, F = 38.193 & p < 0.001, Figure 3C). The elevated expression of miR-2942 by knock-in with mimic did not show any significant increase in eclosion rate (p = 0.314). This signifies an important role played by miR-2942 expression in normal eclosion events in *Ae. albopictus* larvae.

A significant difference is also observed between negative control and normal group (p = 0.004), which suggests that there is a considerable effect in larval development and eclosion after larvae are subjected to microinjection. The method of delivery of exogenous genes by microinjection of larvae in the same way as RNAi in adults is not highly recommended due to possible unavoidable injuries and dilution of injection solution. The larvae could not be subjected to complete drying before microinjection and had to be transferred to water immediately after microinjection. Recently, a new approach for introducing extraneous double stranded RNA for RNA interference and silencing has been established in *Anopheles* larvae by feeding them with nanoparticles conjugated with RNA which has showed better results than conventional microinjection in mosquito larvae [37].

### miR-1891 intervention in *Ae.albopictus* adults

**Figure 4:**
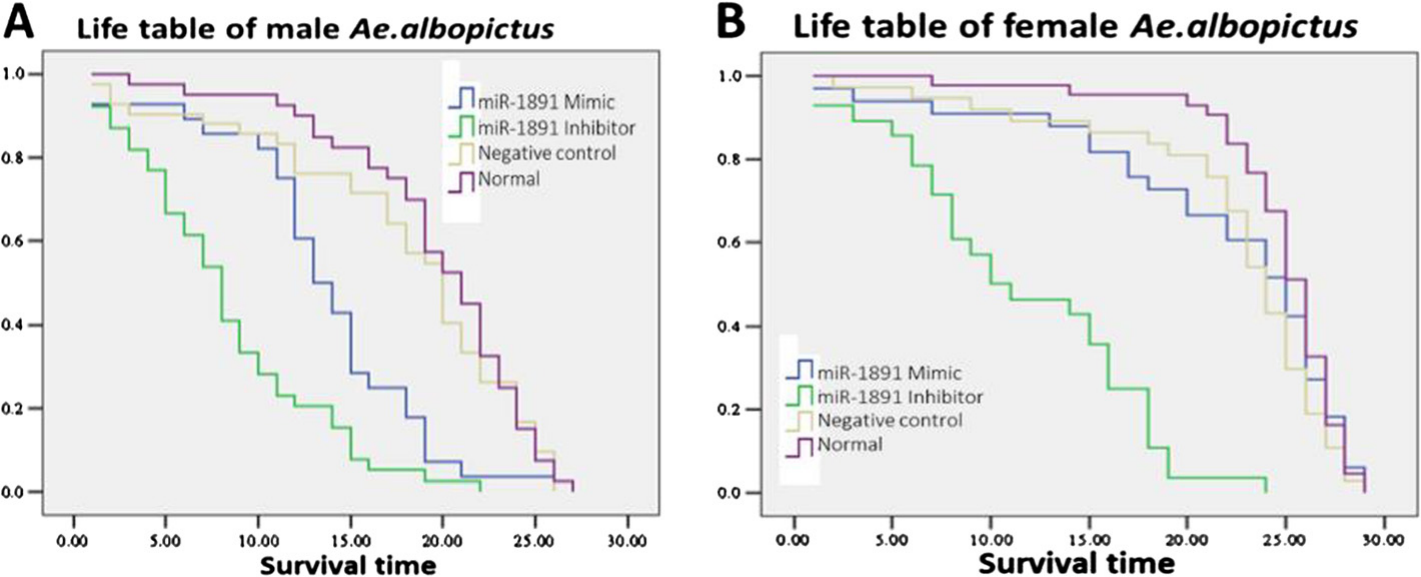
**Longevity (Life table) of male and female adult *Ae. albopictus *after miR-1891 intervention. (A)** Life table of male *Ae. albopictus*. A significant decrease in survival ratio was shown after miR-1891 inhibitor injection in adults (p < 0.001). Statistical analysis by Kaplan-Meier showed X^2^ =33.326, p < 0.05 **(B)** Life table of female *Ae. albopictus*. Statistical analysis by Kaplan-Meier showed X^2^ = 4.274, p < 0.05.

The effect of miR-1891, on longevity after knock-in and knock-down with miRNA mimic and inhibitor in both male and female *Ae. albopictus,* were recorded separately for each group and compared with the control groups (Figure 4). Normally, *Ae. albopictus* under laboratory conditions have a life span of 20–22 days for males and 28–30 days for females. Here we have observed a significant decrease in the life span of both female and male adults in the miR-1891 inhibitor group compared to mimic and control groups (p < 0.001). The mean survival time of males decreased from 16.5 days in the mimic group to 11.33 days and the mean survival time in females reduced from 24.75 days to 13.35 days when miR-1891 expression was knocked-down with the inhibitor.

No significant difference in the mean survival time of two control groups (injected with negative control and un-injected normal control group) were observed (p = 0.058) in male and female mosquitoes indicating that the technique of microinjection of adults did not show any deleterious effect in the normal life span of adults.

The effect of miR-1891 expression in *Ae. albopictus* was also carried out by measuring their influence on the fecundity of the adult mosquitoes. Normally female *Ae. albopictus* lay about 150 to 250 eggs per oviposition. There are 1 to 4 ovipositions per female. But in our laboratory conditions, we have analyzed the eggs laid in their first oviposition to record their fecundity, as the first oviposition is considered a more important measure of fecundity. 72 hours after the males and females were subjected to microinjection with miR-1891mimic, inhibitor and negative control, females were blood fed and the eggs laid were calculated in each group (Figure 3D). The experiments were repeated at different time points for the validation of the result, which was analyzed by ANOVA (F = 58.886 and p < 0.001). The inhibitor group showed a marked decrease in their fecundity which is significantly different from mimic and control groups (p < 0.001). The mean number of eggs laid per female was reduced from 80.35 in normal adults to 23.68 in adults injected with miR-1891 inhibitor, which was significantly less (p < 0.001) compared to all other groups. There was no significant difference among the other three groups. This indicates the possible role played by miR-1891 in the fecundity and development of ovulation or blood meal digestion in female mosquitoes which is important for embryogenesis [38].

## Conclusion

Several miRNAs have recently being categorized in the Asian vector of Dengue fever, *Ae. aegypti*, and it was found that some miRNAs are evolutionarily conserved in the closely related species, *Ae. albopictus*. Stage specific expression of three miRNAs, miR-286b, miR-2942 and miR-1891, which are up-regulated in *Ae. albopictus* were further evaluated in this study to explore their importance in the development of this mosquito species.

The over expression of miRNAs using the specifically designed miRNA mimics based on the endogenously expressed miRNAs, has shown increased levels of expression of miRNA compared to their normal controls. This is confirmed by qRT-PCR, both by *in vitro* expression in C6/36 mosquito cell lines after transfection as well as in *in vivo* expression in developmental stages of mosquitoes by microinjection. The increased expression of these miRNAs in their specific developmental stages did not show any enhanced functional role in their normal development. But the knock down of expression of these endogenously expressed miRNAs with the corresponding inhibitors has shown considerable decrease in the expression levels of these miRNAs and obvious functional effects in the development of *Ae. albopictus*.

Upon miRNA inhibition of embryo specific miR-286b in embryos, there was a considerable reduction in hatching rate and also a noticeable delay in hatching of the embryos. In larvae, on inhibition of expression of larval specific miRNA, miR-2942, a reduced eclosion rate was observed which was significantly different from the mimic group. A significant difference is also observed between negative control injected group and normal un-injected group. This result indicates that there is a considerable effect of larval development and eclosion after it is subjected to microinjection.

*Ae. albopictus* adult specific miRNAs, miR-1891, when knocked down, has shown a significant reduction in the life span (longevity) and also in a marked reduction in fecundity (egg laying capacity). This indicates a possible regulatory role of miR-1891 in the fecundity and development of ovulation or blood meal digestion in female mosquitoes. These results are a positive indication of the important role played by these mosquito-specific miRNAs and their temporal and stage-specific up-regulation in the developmental cycle of *Ae. albopictus*. Further analysis of the regulatory mechanisms at the transcriptome level will be possible after predicting their target proteins and performing interventional studies on the target mRNA expression in *Ae. albopictus* after its genome sequence is available.

The knock-down and loss of function of endogenously expressed miRNAs by the miRNA inhibitors in specific developmental stages of *Ae. albopictus* has shown considerable effects in the development, but enhancement of their gain of function was not observed on knock-in of these specific miRNAs. This clearly demonstrates that these specifically and temporally up-regulated miRNAs in various developmental stages of *Ae. albopictus* have important regulatory functions to play in their normal development and vital functions like longevity and fecundity which are important for the vectorial population density and pathogen transmissibility of this mosquito vector. The miR-mimics corresponding to all three miRNAs did not show any elevated functional developmental parameters and remain constant relative to negative control groups. This possibly indicates a saturation level being maintained by the endogenous miRNA expression specific to their gain of function. The exogenously introduced miRNA mimic exhibited no functional effect when they were specifically knocked-in. The result of the miRNA interventional experiments on functional analysis clearly indicates that an optimal level of endogenous expression of miRNA is maintained and is indispensable for the normal functioning and their further development in the *Ae. albopictus* life cycle.

Our study presents for the first time a comprehensive analysis of *Ae. albopictus* specific miRNAs and their role in regulating the stage-specific development of this important mosquito vector species in China and in many Southeast Asian countries. This research finding established a new platform for carrying out further detailed ongoing studies to delineate the exact functional roles played by these miRNAs at the transcriptomic and genomic level. Further ongoing research work needs to be established after the genome sequence is available for this mosquito species to find out the transcriptomic level of the miRNA's regulatory role in many of these phenotypic changes in the developmental stages of this mosquito species, including whether the miRNA influence the maternal or paternal gene regulation to affect fecundity after miRNA intervention. Further prospects associated with this work, including the target specific functional studies, may open up many new avenues in mosquito control strategies.

## Competing interests

The authors declare that they have no competing interests.

## Authors’ contributions

XC and SPK conceived and designed the experiments. SPK and YY made all sample collection, performed the experiments, analyzed the data and wrote the initial draft. YL helped in computation of results and statistical analysis. JG, HP & XC intellectually supported the study and corrected the manuscript drafts. All authors read and approved the final manuscript.

## Supplementary Material

Additional file 1 Sequences of three mature aae-miRNAs retrieved from the miRBase with their accession numbers. Click here for file

Additional file 2 **The sequences of miRNA mimic, inhibitor and negative control of three miRNAs, miR-1891, miR-286b and miR-2942.** The mimics are double stranded RNAs and inhibitors are single stranded.
Click here for file

Additional file 3 **Image of the agarose gel electrophoresis of the total RNA extracted from C6/36 cells after transfection with miRNA mimics, inhibitors and negative controls.** 1% agarose gel loaded with 3 μl each of the extracted RNA samples loaded in each well as marked and electrophoresis carried out at 100 V constant voltage for 25 minutes.
Click here for file

Additional file 4 **The agarose gel electrophoresis of the total RNA extracted from *Ae. albopictus *embryo, larvae and adults after injection with miRNA mimics, inhibitors and negative controls.** 1% agarose gel loaded with 3 μl each of the extracted RNA samples in each well as marked and run for 25 minutes.
Click here for file
