# Supplementary figures and images for: Functional characterization of three MicroRNAs of the Asian Tiger Mosquito, Aedes albopictus
Source: Parasit Vectors. 2013 Aug 8;6:230. doi: 10.1186/1756-3305-6-230 (PMC3750763; doi:10.1186/1756-3305-6-230)

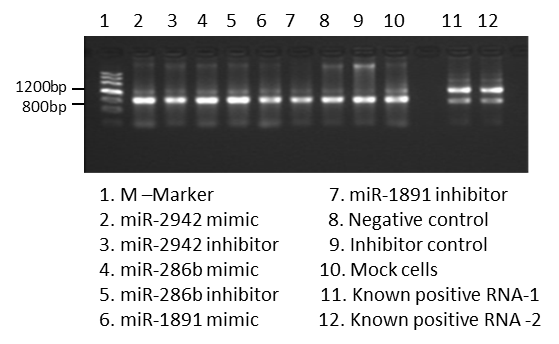

Supplement: Additional file 3 — Image of the agarose gel electrophoresis of the total RNA extracted from C6/36 cells after transfection with miRNA mimics, inhibitors and negative controls. 1% agarose gel loaded with 3 μl each of the extracted RNA samples loaded in each well as marked and electrophoresis carried out at 100 V constant voltage for 25 minutes. [file 1756-3305-6-230-S3.tiff]

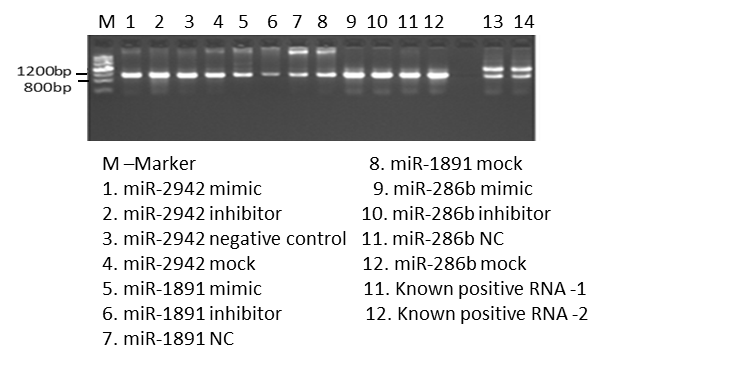

Supplement: Additional file 4 — The agarose gel electrophoresis of the total RNA extracted from Ae. albopictus embryo, larvae and adults after injection with miRNA mimics, inhibitors and negative controls. 1% agarose gel loaded with 3 μl each of the extracted RNA samples in each well as marked and run for 25 minutes. [file 1756-3305-6-230-S4.tiff]
